# Adjunctive Corticotherapy for Community Acquired Pneumonia: A Systematic Review and Meta-Analysis

**DOI:** 10.1371/journal.pone.0144032

**Published:** 2015-12-07

**Authors:** Christophe Marti, Olivier Grosgurin, Stephan Harbarth, Christophe Combescure, Mohamed Abbas, Olivier Rutschmann, Arnaud Perrier, Nicolas Garin

**Affiliations:** 1 Division of General Internal Medicine, Department of Internal Medicine, Rehabilitation and Geriatrics, Geneva University Hospitals and Geneva Faculty of Medicine, Geneva, Switzerland; 2 Division of Infectious Diseases, Department of Medical Specialties, Geneva University Hospitals and Geneva Faculty of Medicine, Geneva, Switzerland; 3 Department of Health and Community Medicine, University Hospitals of Geneva and Geneva Faculty of Medicine, Geneva, Switzerland; 4 Division of Emergency Medicine, Geneva University Hospitals and Geneva Faculty of Medicine, Geneva, Switzerland; 5 Division of Internal Medicine, Hôpital Riviera-Chablais, Monthey, Switzerland; San Raffaele Scientific Institute, ITALY

## Abstract

**Background:**

Community-acquired pneumonia (CAP) induces lung and systemic inflammation, leading to high morbidity and mortality. We systematically reviewed the risks and benefits of adjunctive corticotherapy in the management of patients with CAP.

**Methods:**

We systematically searched Pubmed, Embase and the Cochrane Library for randomized controlled trials comparing adjunctive corticotherapy and antimicrobial therapy with antimicrobial therapy alone in patients with CAP. The primary outcome was 30-day mortality. Secondary outcomes were length of hospital stay, time to clinical stability and severe complications.

**Results:**

14 trials (2077 patients) were included. The reported 30-day mortality was 7.9% (80/1018) among patients treated with adjunctive corticotherapy versus 8.3% (85/1028) among patients treated with antimicrobial therapy alone (RR 0.84; 95%CI 0.55 to1.29). Adjunctive corticotherapy was associated with a reduction of severe complications (RR 0.36; 95%CI 0.23 to 0.56), a shorter length of stay (9.0 days; 95%CI 7.6 to 10.7 vs 10.6 days; 95%CI 7.4 to 15.3) and a shorter time to clinical stability (3.3 days; 95% CI 2.8 to 4.1 vs 4.3 days; 95%CI 3.6 to 5.1). The risk of hyperglycemia was higher among patients treated with adjunctive corticotherapy (RR 1.59; 95%CI 1.06 to 2.38), whereas the risk of gastro-intestinal bleeding was similar (RR 0.83; 95%CI 0.35 to 1.93). In the subgroup analysis based on CAP severity, a survival benefit was found among patients with severe CAP (RR 0.47; 95%CI 0.23 to 0.96).

**Conclusion:**

Adjunctive corticotherapy is associated with a reduction of length of stay, time to clinical stability, and severe complications among patients with CAP, but the effect on mortality remains uncertain.

## Introduction

Lower respiratory infections, including community-acquired pneumonia (CAP), are the second cause of years of life lost worldwide, and the 9^th^ cause in Western Europe.[1] Annual incidence of hospitalization for CAP is 2.75 to 2.96 per 1000 with an in hospital mortality of 14%.[2] Mortality rises to 27% when intensive care unit admission is needed.[3] CAP can induce severe lung and systemic inflammation, and is the most frequent cause of severe sepsis and acute respiratory distress syndrome.[4–5] High inflammatory cytokines levels are associated with an increased risk of both early and late death in CAP.[6–7] Moreover, historical studies have shown that early mortality of bacteremic pneumococcal CAP is not affected by penicillin treatment.[8] A detrimental effect of the host inflammatory response might explain the failure of antibiotics to impact early clinical course of CAP. This suggests that modifying the inflammatory response is required to improve the persistently severe prognosis of this infection.

Corticosteroids are among the most potent anti-inflammatory drugs available. Use of corticosteroids is beneficial in other infectious diseases, such as bacterial meningitis or septic shock.[9–10] Moreover, corticosteroids have been shown to improve oxygenation and reduce the need for vasopressors by damping lung inflammation in acute respiratory distress syndrome.[5] Randomized controlled trials (RCT) of adjunctive corticotherapy in CAP have generally resulted in a modest improvement of clinical and physiological parameters and a reduced length of stay.[11–13] Previous meta-analyses suggested a possible mortality benefit restricted to patients with severe CAP.[14–15] Nevertheless, confidence intervals of estimates in these studies were wide due to the limited number of patients in the included studies. Two clinical trials have been recently published, and were not included in these meta-analyses. With 785 patients included, the STEP trial is the largest RCT investigating adjunctive corticotherapy in CAP.[16] The main findings of this trial were a shorter time to clinical stability and shorter length of stay in the corticosteroid arm. Severe events were infrequent and did not differ between the two arms. In another RCT, Torres et al. included 120 patients with severe CAP and a C-reactive protein of more than 150 mg/L.[17] They found a lower rate of late treatment failure in the arm receiving adjunctive corticotherapy, an advantage driven predominantly by a lower rate of radiographic progression. However, other outcomes, including time to clinical stability or length of stay, did not differ between the two arms.

In view of these recent trials, and considering the lack of unequivocal conclusions concerning a benefit of adjunctive corticotherapy on hard clinical endpoints, we conducted an updated systematic review and meta-analysis to better evaluate the advantages and risks of adjunctive corticotherapy in CAP.

## Materials and Methods

Search strategy, study selection, data extraction and analysis were performed according to a pre-defined protocol (available on request) and according to the PRISMA guidelines[18] (S1 File).

### Search strategy

Two authors (CM, OG) systematically searched Medline, Embase and the Cochrane Controlled Trials registry using the following key words without language restriction: (*Community acquired pneumonia* AND [*corticosteroids* OR corticotherapy *OR steroids OR dexamethasone OR prednisone OR cortisone OR hydrocortisone OR prednisolone*]). The detailed search strategy is available in the supplementary appendix (S1 File). To ensure a comprehensive literature search, we examined reference lists from retrieved articles and reference literature (guidelines and systematic reviews) and questioned experts in CAP for possible published or unpublished missing studies.

### Study selection and data extraction

We included randomized controlled trials comparing antimicrobial therapy and adjunctive systemic corticotherapy with antimicrobial therapy alone in adult patients with CAP of any severity. Studies including paediatric populations, nosocomial pneumonia, viral pneumonia only, or comparing two regimens of corticotherapy were excluded. Two investigators (CM, OG) independently evaluated studies for possible inclusion. Non-relevant studies were excluded based on title and abstract. For potentially relevant studies, full-text was obtained and two investigators (CM, NG) independently assessed study eligibility and extracted the data on study design, patient characteristics and outcomes. Disagreement about study inclusion or data extraction was resolved by consensus or by discussion with a third author (OG).

### Outcomes and measurements

The primary efficacy outcome was 30-day all-cause mortality. Five secondary efficacy outcomes were considered: length of hospital stay (LOS), time to clinical stability (TCS), need for vasopressors, need for mechanical ventilation (invasive or non invasive) and severe complications (need of mechanical ventilation or vasopressors). Two safety outcomes were considered: risk of hyperglycemia (proportion of patients requiring new insulin therapy) and risk of in-hospital gastro-intestinal bleeding. Since trials were performed over more than 50 years, we accepted The British Thoracic Society criteria [19] and the successive American Thoracic Society criteria[20–21] to define severe CAP. Corticosteroid doses were converted to prednisone equivalents with a web-based convertor.[22]

### Study quality assessment

Quality of included studies was assessed using the criteria developed by Jadad et al.[23] evaluating the quality of randomization, blinding and handling of exclusion and attrition. Two investigators (CM, NG) assessed study quality independently. Disagreements were resolved by consensus.

### Data analysis

All analyses were performed on data reported according to the *intention to treat* principle. For each dichotomous outcome and for each study, 2x2 tables summarizing the number of patients experiencing the outcome in each group and the number of patients at risk were constructed. Treatment effects were expressed as Risk Ratios (RR) and were pooled over studies by using the Mantel-Haenszel method given the small sample size in some studies and the low prevalence of events.[24] A continuity correction of 0.5 was applied for studies without event in one arm. Random effect models were used throughout. For statistically significant results, the number needed to treat (NNT) was derived from the combined RR and the combined prevalence of the outcome in the control arm. Continuous outcomes were found log-normally distributed by reconstructing individual data from provided Kaplan-Meier curves and comparing the latter with survival curves fitted with a parametric model assuming a log-normal distribution of the time-to-event. In this situation, it is recommended to combine the means of logarithm of outcomes rather than the means of outcomes [25] Means of logarithm were derived from logarithm of medians and inter-quartile ranges or from means and standard deviation following the methods proposed by Wan et al.[26] and by Higgins et al.[25] Authors of the original studies were solicited to complete outcomes if missing. Differences in means of logarithm of outcomes were combined using models with random effects (Der Simonian and Laird’s method) and the overall treatment effects were expressed as Geometric Means Ratios (GMRs). The significance level was set at 0.05. The heterogeneity was measured by the I^2^ statistic.[27] For the outcome mortality, potential heterogeneity factors were explored by pre-specified subgroup analyses: patients with severe CAP (according to the British Thoracic Society or American Thoracic Society criteria); and the duration and dose of corticosteroids (more than 5 days versus 5 days or less, more than 50 mg/day of prednisone equivalent or 50 mg/day or less). Finally, we performed a metaregression to explore a potential variation of the risk ratios over time using the logarithm of the risk ratios over years of publication. To evaluate the impact of treatment dose and duration on treatment effect estimate, we performed univariate and multivariate regressions in a model including treatment dose (inferior or equal to prednisone 50mg equivalent or more), duration (0 to 5 days or > 5 days) and CAP severity.

Sensitivity analyses were conducted to check the robustness of the pooled RRs by removing each study one-by-one by excluding older studies conducted in the seventies or before, and by excluding studies with higher risk of bias (Jadad score inferior or equal to 3). Additionally, in order to account for studies without an event in both arms, an exact method [28] was applied. Publication bias was assessed using inspection of the funnel plot, Egger’s test and the trim and fill method.[29] The R packages “meta: Meta analysis with R, version 1.6–1” and “exactmeta: Exact fixed effect meta analysis, version 1.0–2”were used for these analyses.

## Results

### Study selection and characteristics

**Fig 1:**
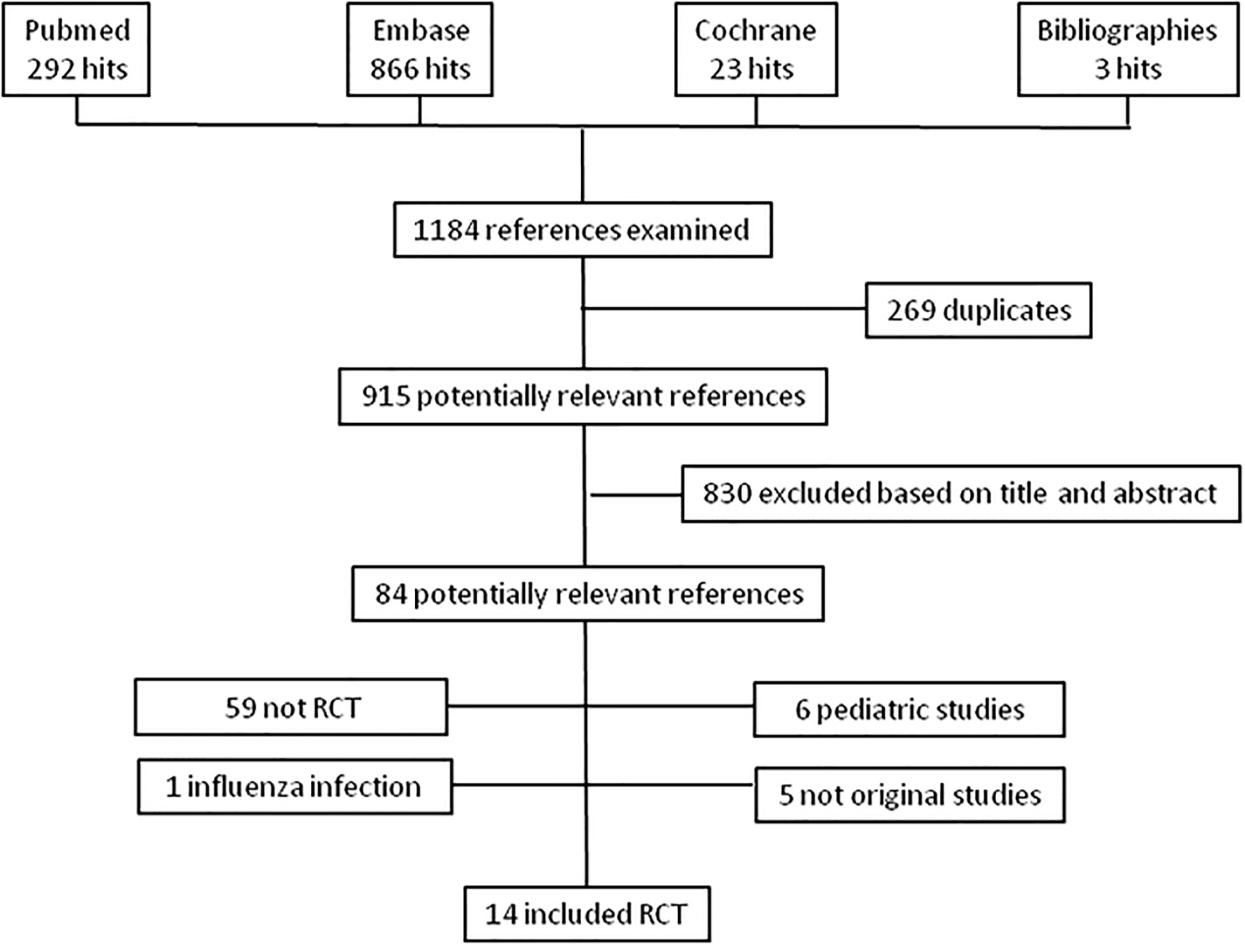
Study Flow Chart.

The search retrieved a total of 1184 references, among which 269 duplicates were identified. Of the remaining 915 articles, 830 were excluded based on title and abstract (Fig 1). Full text was obtained for the remaining 84 references. Of these, 5 did not contain original data, 59 were not randomized control trials, 6 included pediatric patients, 1included viral pneumonia only and 14 satisfied inclusion criteria.[11, 13, 16, 30–40] Five studies [11, 13, 30, 36, 40] included patients with severe CAP and 9 studies patients with mild to severe CAP.[16, 31–32, 34–35, 37–39] The median daily prednisone equivalent and treatment duration were 45mg and seven days in available studies. The main characteristics of the included studies are displayed in Table 1.

**Table 1:** Characteristics of included studies.

1st author	Year	Inclusion criteria	Diagnostic criteria	Microbiologically confirmed (%)	Number included	Active treatment (dose mg)	Mean prednisone equivalence (mg/d)	Treatment duration (days)	Study Quality Assessment*		
									Randomisation	Blinding	Attrition
Bennett [38]	1963	Severe infections	NA	100	49	Hydrocortisone (300) degressive	44	6	2	2	1
Blum [16]	2015	CAP	x-ray+clinical	23.9	785	Prednisone (50)	50	7	2	2	2
Confalonieri [11]	2004	SCAP (ATS 1993)	x-ray +ATS	65.2	48	Hydrocortisone (2oo) mg iv then 10mg/h/7d	67	7	2	2	1
Fernández-Serrano [13]	2011	SCAP (PO2/FIO2 and multilobar)	x-ray+clinical	80	56	Methylprednisone (200) then 20mg/6h degressive + omeprazole	86	9	1	2	2
Klastersky [39]	1971	"Life-threatening infection"	NA	NA	42	bethametasone (1mg/kg)	525	3	2	1	1
Marik [30]	1993	SCAP (BTS criteria)	BTS criteria	66.7	30	Hydrocortisone (10 mg/kg)	17.5	single dose	2	1	1
McHardy [31]	1972	CAP	x-ray + clinical	70.6	126	Prednisolone (4x5/d)	20	7	1	0	1
Meijvis [32]	2011	CAP	x-ray+clinical	57.6	304	dexamethasone (5)	31	4	2	2	2
Mikami [33]	2007	CAP	x-ray+clinical	41.9	31	Prednisolone (40)	40	3	1	0	2
Nafae [34]	2013	CAP	x-ray+clinical	NA	80	hydrocortisone (200mg iv, then 10mg/h/7d)	67	7	1	0	2
Sabry [40]	2011	SCAP (ATS 2001)	x-ray+clinical	NA	80	Hydrocortisone (300mg iv then 12.5mg/h/7d)	86	7	0	0	2
Snijders [35]	2010	CAP	x-ray+clinical	55.4	213	Prednisolone (40)	40	7	2	2	2
Torres [36]	2015	SCAP (ATS 2007); CRP >150	x-ray+clinical	40.8	120	methylprednisone (1 mg/kg)	88	5	2	2	2
Wagner [37]	1955	pneumococcal CAP	NA	100	113	hydrocortisone (80)	20	5	1	1	2

CAP: Community acquired Pneumonia, SCAP: Severe Community acquired Pneumonia, ATS: American Thoracic Society, BTS: British Thoracic Society, CRP: C-reactive protein *Randomisation: described as randomised 1 point, adequate randomisation method (concealment) 1 additional point, Blinding: described as blinded 1 point, adequate blinding, 1 additional point, Attrition: description of exclusion (1 point) and withdrawals (1 additional point)

### Study quality and risk of bias

Among the 14 included studies, 10 were considered of good quality (Jadad score 4–6), three of fair quality (Jadad score 3) and one of poor quality (Jadad score 1–2). The quality of randomization was considered adequate in eight studies (Table 1). Both patients and investigators were blinded to the treatment arm in seven studies. One study [11] was stopped prematurely because of a benefit of the intervention arm which may represent a potential source of bias. Diagnosis of pneumonia was based on clinical history and chest X-ray or BTS criteria in eleven studies but not specified in three older studies.

### 30-day mortality

**Fig 2:**
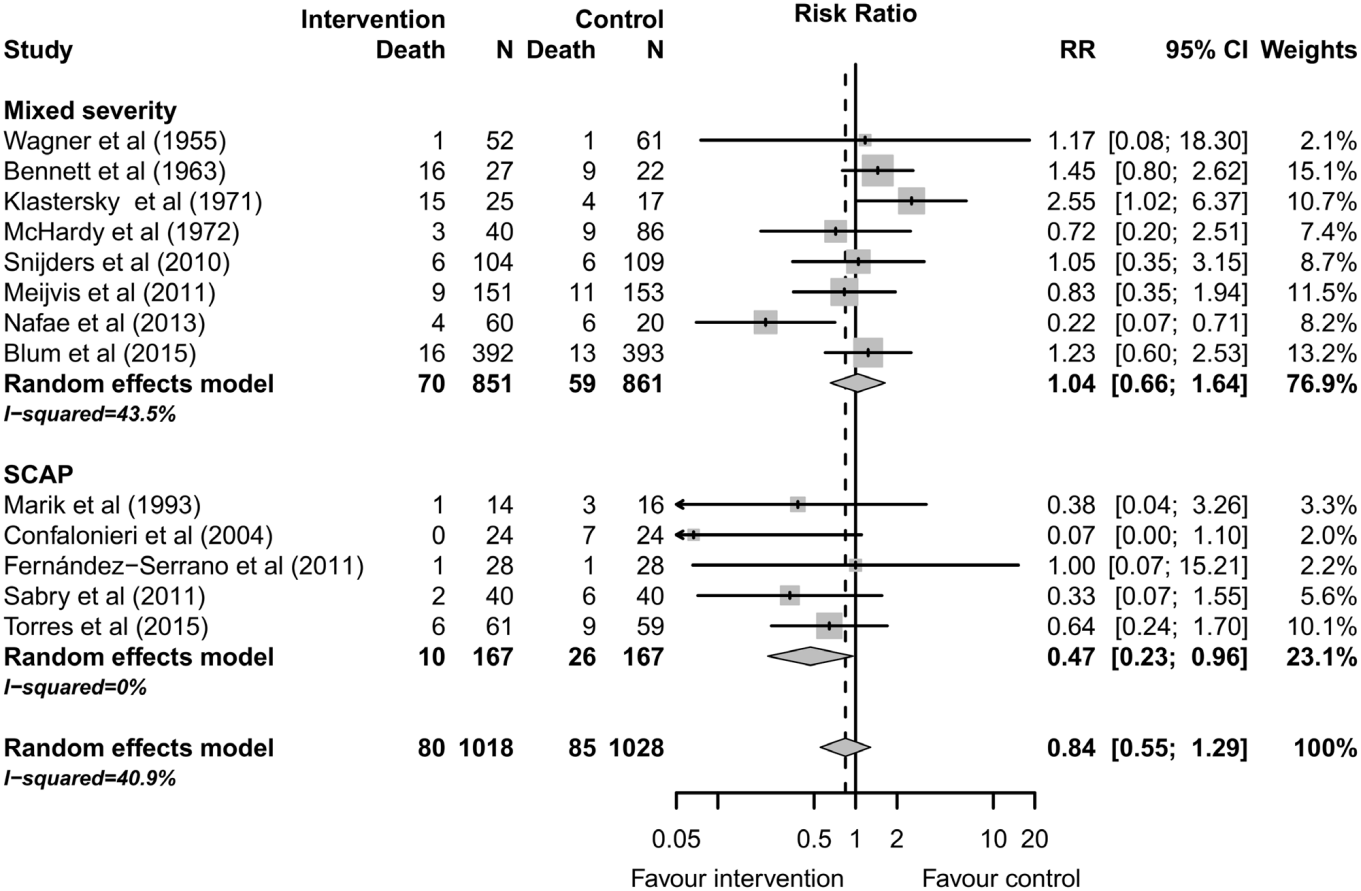
30-day Mortality according to CAP severity.

Thirteen studies including 2046 patients reported 30-day mortality.[11, 13, 16, 30–32, 34–40] The reported mortality was 7.9% (80/1018) among patients treated with adjunctive corticotherapy versus 8.3% (85/1028) among patients treated with antimicrobial therapy alone (RR 0.84; 95% CI 0.55 to 1.29, p = 0.42). Moderate heterogeneity was observed among studies (I^2^ = 40.9%). (Fig 2 and Table 2)

**Table 2:** Main results for binary outcomes (Mantel-Haenszel method, random effect model).

Outcome	Nb studies	Pooled RR (95%CI)	p-value	I^2^	NNT (95%CI)
**Mortality**	13	0.84 (0.55 to 1.29)	0.4296	40.9%	NA
**Mortality (severe CAP studies)**	5	0.47 (0.23 to 0.96)	0.0380	0.0%	11 (7 to 144)
**GI Bleeding**	9	0.83 (0.35 to 1.93)	0.6633	0.0%	NA
**Hyperglycemia**	8	1.59 (1.06 to 2.38)	0.0248	29.9%	24 (10 to 236)
**Mechanical ventilation**	7	0.41 (0.29 to 0.60)	<0.0001	0.0%	7 (6 to 11)
**Needs Vasopressor**	4	0.33 (0.10 to 1.17)	0.0847	25.2%	NA
**Severe complications**	4	0.36 (0.23 to 0.56)	<0.0001	0.0%	4 (3 to 6)

A risk ratio higher than 1 means that the risk is greater in intervention arm than in control arm.NA: Not applicable, RR:relative risk, NNT: Number needed to treat

Five studies [11, 13, 30, 36, 40] including 334 patients reported mortality for patients with severe CAP. The reported mortality was 6.0% (10/167) among patients treated with adjunctive corticotherapy versus 15.6% (26/167) among patients treated with antimicrobial therapy alone (RR 0.47; 95% CI 0.23 to 0.96, p = 0.038). No heterogeneity was observed among studies including severe CAP (I^2^ = 0%), (Fig 2).

### Secondary Outcomes

#### Length of stay

**Fig 3:**
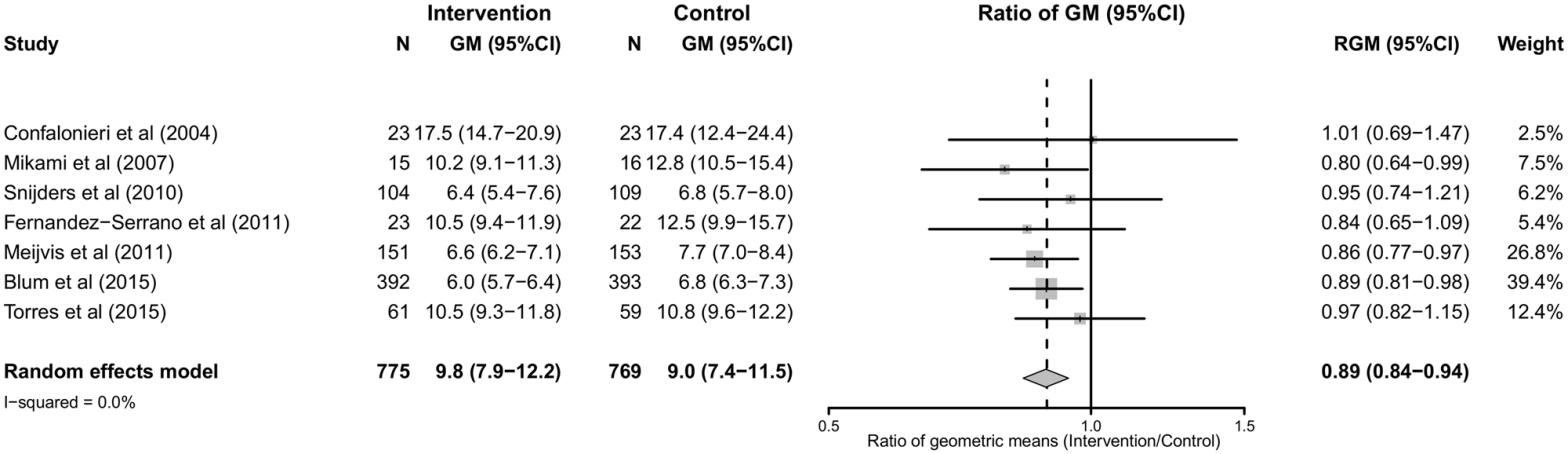
Length of hospital stay (LOS), forrest plot.

Eight studies including 1624 patients reported on LOS.[11, 13, 16, 32–33, 35–36] The pooled geometric mean of LOS was 9.0 days (95% CI 7.6 to 10.7) for patients treated with adjunctive corticotherapy versus 10.6 days (95%CI 7.4 to 15.3) for patients treated with antimicrobial therapy alone (geometric mean ratio 0.82; 95% CI 0.73 to 0.91, p = 0.0004). High heterogeneity was observed among studies (I^2^ = 97%). (Fig 3 and S2 File)

#### Time to clinical stability

Four studies including 1163 patients reported TCS.[13, 16, 35–36] The pooled geometric mean of TCS was 3.3 days (95% CI 2.7 to 4.1) for patients treated with adjunctive corticotherapy versus 4.3 days (95% CI 3.6 to 5.1) for patients treated with antimicrobial therapy alone (geometric mean ratio 0.79; 95% CI 0.70 to 0.89, p = 0.0005). Moderate heterogeneity was observed among studies (I^2^ = 35.5%). (S2 File)

#### Severe Complications

Four studies including 304 patients reported on severe complications (need for vasopressors or mechanical ventilation).[11, 13, 34, 36] The reported risk of severe complications was 12.1% (21/173) among patients treated with adjunctive corticotherapy versus 37.4% (49/131) among patients treated with antimicrobial therapy alone (RR 0.36; 95% CI 0.23 to 0.56, p<0.0001) (S2 File). No heterogeneity was observed among studies (I^2^ = 0%)

Four studies including 266 patients reported on the need for vasopressors.[11, 13, 36, 39] The reported need for vasopressors was 3.6% (5/138) among patients treated with adjunctive corticotherapy versus 14.5% (20/128) among patients treated with antimicrobial therapy alone (RR 0.33; 95% CI 0.10 to1.16, p = 0.08), (S2 File). Moderate heterogeneity was observed among studies (I^2^ = 25.2%).

Seven studies including 1199 patients reported on the need for mechanical ventilation.[11, 13, 16, 30, 34, 36, 40] The reported need for mechanical ventilation was 5.3% (33/619) among patients treated with adjunctive corticotherapy versus 12.1% (70/580) among patients treated with antimicrobial therapy alone (RR 0.41; 95% CI 0.29 to 0.60, p<0.0001), (S2 File). No heterogeneity was observed among studies (I^2^ = 0%).

#### Hyperglycemia

Hyperglycemia was reported in eight studies including 1619 patients.[13, 16, 30, 32–36] The reported proportion of patients requiring insulin therapy was 15.4% (127/825) among patients treated with adjunctive corticotherapy versus 8.2% (65/794) among patients treated with antimicrobial therapy alone (RR 1.59; 95% CI 1.06 to 2.38, p = 0.025). Moderate heterogeneity was observed among studies (I^2^ = 29.9%) (S2 File).

#### Gastro-intestinal bleeding

Nine studies including 1616 patients reported on gastro-intestinal bleeding.[11, 13, 16, 30, 32, 34, 36–37, 40] The reported risk for gastro-intestinal bleeding was 1.1% (9/822) among patients treated with adjunctive corticotherapy versus 1.3% (10/794) among patients treated with antimicrobial therapy alone (RR 0.83; 95% CI 0.35 to1.93, p = 0.66). No heterogeneity was observed among studies (I^2^ = 0%), (S2 File).

### Sources of heterogeneity and sensitivity analyses

Heterogeneity among studies was moderate for the primary outcome (I^2^, 40.9%) and low for binary secondary outcomes (I^2^ inferior to 30%, Table 2.) For the primary outcome, CAP severity was a potential source of heterogeneity. Accordingly, no heterogeneity was detected among studies including severe CAP only. The meta–regression of the treatment effect estimate according to CAP severity was close to statistical significance (p = 0.067, S3 File). Among studies including mixed severity CAP, the study by Nafae et al.[34] contributed to the inter-study heterogeneity and no heterogeneity was present after exclusion of this study. For continuous outcomes, high heterogeneity was detected for LOS but was mainly explained by the study by Nafae et al. No heterogeneity was observed after deletion of this study. Nevertheless, no significant change in treatment estimate was observed after deletion of this study (S3 File).

No association between treatment duration or daily dose of corticosteroids was detected in our subgroup analyses and meta-regression. The sensitivity analysis using the exact method model yielded estimates similar to those of the random Mantel-Haenszel model. Excluding studies one by one did not significantly alter the treatment estimates with regard to the overall mortality, but statistical significance was lost after exclusion of single studies [11, 30, 40] in the subgroup of severe CAP studies (S3 File). Similarly, exclusion of older studies [31, 37–39] did not significantly alter the treatment estimates with regard to the overall mortality (S3 File) .Studieswith higher risk of bias tended to overestimate treatment benefits and underestimate treatment harms, but the difference was statistically significant only for the outcome hyperglycemia (S3 File). Finally the metaregression of treatment effect estimate according to the year of publication showed a positive relationship between treatment estimate and year of publication that was close to statistical significance (p = 0.063, S3 File)

### Publication bias

**Fig 4:**
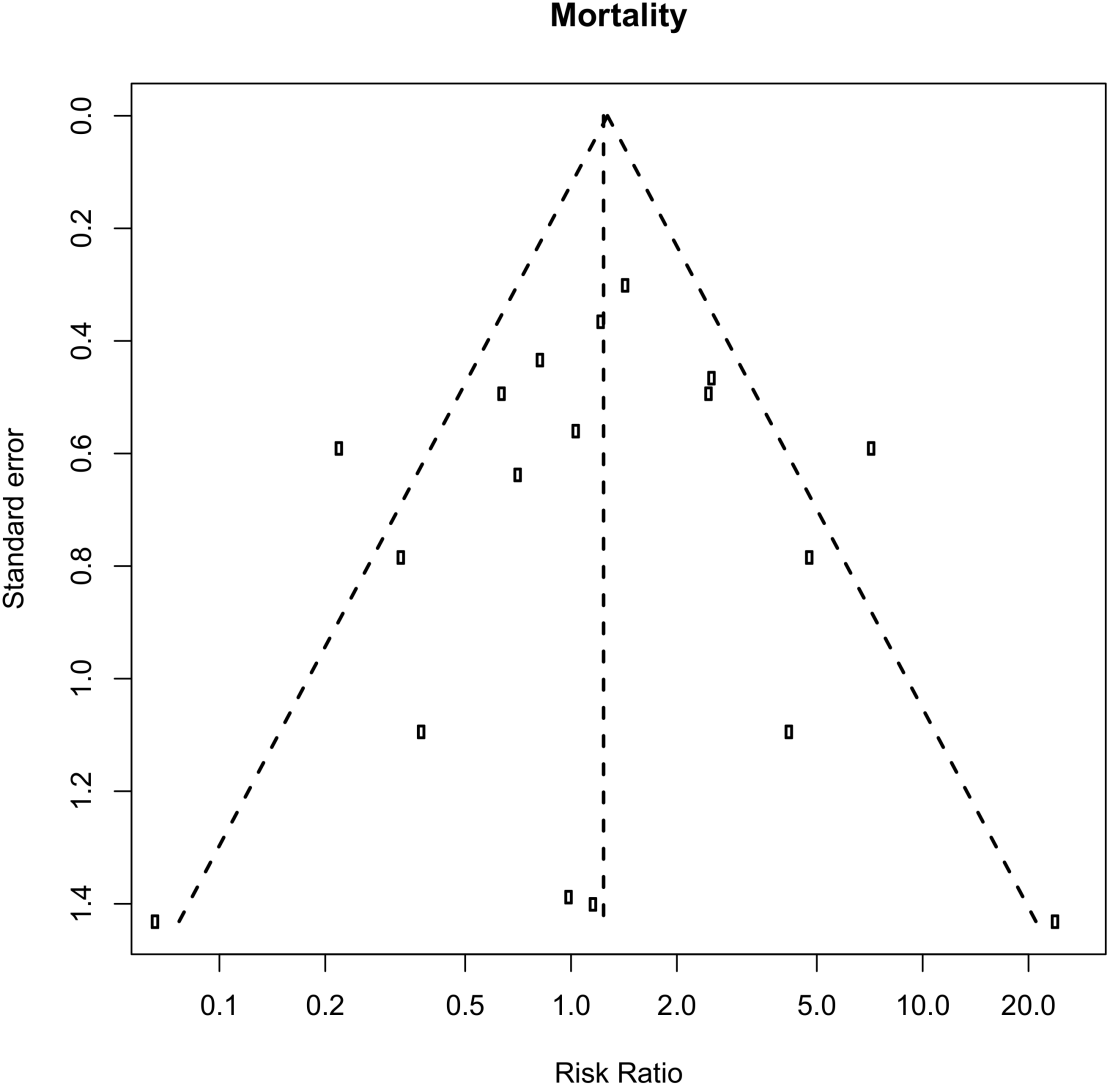
Evaluation of potential publication bias for 30-day Mortality, funnel plot.

Inspection of the funnel plots was suggestive of potential publication or reporting bias for most of the outcomes (S4 File). The funnel plot for mortality showed slight asymmetry (Fig 4). However, Egger’s test did not indicate significant publication bias (P = 0.37) for 30-day mortality. After correction of potential publication bias using the Trim & Fill method, the pooled RRs were not modified but statistical significance was lost for the outcome mortality in SCAP studies (RR 0.54; 95% CI 0.27 to 1.08, p = 0.0815), (S4 File).

## Discussion

In the present systematic review including more than 2000 patients, adjunctive corticotherapy is associated with a reduced risk of severe complications, a reduced LOS and a shorter TCS in patients with CAP. These benefits are robust and not altered by sensitivity analyses or the correction of potential publication bias. In contrast, no benefit in 30-day mortality was observed among all patients. When analyzing studies with mixed-severity disease and severe CAP separately, mortality was significantly reduced in the subgroup of patients with severe CAP. Nevertheless, the confidence interval around treatment estimate was wide for this outcome due to the relative small number of events and this mortality benefit was sensitive to correction of potential publication bias or deletion of single studies. Moreover, one of the studies included in this subgroup analysis [11] was interrupted prematurely after an interim analysis showing a mortality reduction among patients receiving corticotherapy which may represent an additional source of bias and overestimate treatment effect.[41]

Previous meta-analyses about corticotherapy in CAP have reported a mortality reduction in patients with severe CAP or CAP related severe sepsis or acute lung injury.[14–15, 42–43] Corticosteroids induce a rapid decrease in inflammatory markers and cytokines,[11, 33]alleviating lung and systemic inflammation, and potentially preventing respiratory and circulatory failure among the most severely ill patients.[14] We observed a two-fold reduction of the need for mechanical ventilation, and a three-fold reduction of the need for vasopressor, although this latter endpoint did not reach statistical significance. Similarly, Annane et al. have shown that glucocorticoid treatment of severe sepsis or septic shock was associated with increased 28-day shock reversal but no clear benefit on mortality.[44] They suggested a possible mortality benefit among studies using prolonged (> 5days) and low-dose glucocorticoid therapy (< 300mg hydrocortisone equivalent). Contrasting with these results, we did not observe any significant interaction between dose and duration of glucocorticoids and treatment effect. The mortality benefit of adjunctive corticotherapy among the most severely ill patients has been further suggested in a large cohort of CAP patients from Japan (n = 6925).[45] In a propensity score-matched analysis, Tagami et al. observed a significant 28-day mortality reduction among CAP patients requiring vasopressor therapy, but not in haemodynamically stable patients. Similarly, in a recently published meta-analysis, Siemieniuk et al. reported an interaction between mortality benefit and CAP severity but no consistency in the subgroup effect with related outcomes such as need for mechanical ventilation.[43] These investigators observed a greater reduction in mortality among patients with severe pneumonia and a greater reduction of mechanical ventilation among patients with less severe illness. We believe that this apparent inconsistency is not surprising since original studies usually did not report separately respiratory failure present at inclusion or developing during follow-up. Indeed, limited benefit is expected for this outcome in studies with a higher proportion of respiratory failure at inclusion.

There are some differences between the recently published study by Siemienuk et al. and our study. First, our litterature search identified two older studies [38–39] reporting outcomes for CAP patients but failed to identify the study by El Ghamrawy et al.[46] Second, we systematically extracted intention-to treat populations and used a statistical model accounting for the log-normal distribution of continuous outcomes. Third, we were able to identify an error in the legend of the largest study about steroids and CAP[16] which reported 95% confidence intervals around treatment effect estimates as interquartile ranges allowing us to use the correct results after confirmation from the authors. Finally we limited the definition of severe pneumonia to the BTS society rule and ATS criteria whereas Siemienuk et al. accepted wider definitions including author’s definitions or control group mortality over 15%. Nevertheless, and despite some slight differences in study severity classification and treatment estimates, our conclusions are similar, mutually strengthening their validity. Similarly, Wan et al recently issued another meta-analysis about corticosteroids in CAP but this study identified fewer studies (9 versus 14) and did not report pooled results for TTCS or LOS:

The lack of apparent mortality benefit among less severe patients deserves several comments; first, mortality is relatively rare among non severe CAP, limiting the statistical power for this outcome. Moreover CAP is a heterogeneous clinical syndrome with diverse viral or bacterial etiologies, and disease severity results from the combination of pathogen and host-associated factors, including co-morbid conditions and inflammatory response. These aspects probably differ between severe CAP and patients with less severe presentation and greater benefit of corticosteroids is expected among patients with a higher inflammatory burden. Moreover, case-control and cohort studies have suggested an increased mortality among patients with viral pneumonia receiving corticosteroids.[47] Unfortunately, a subgroup analysis based on the type of pathogen could not be performed since only a few studies reported outcomes according to microbiologic aetiology. In the largest available study,[16] no interaction between the positivity of blood cultures or the magnitude of the C-reactive protein elevation and treatment effect was detected regarding TCS.

Among patients with less severe disease, down-regulation of inflammatory cytokines by glucocorticoids may lead to faster resolution of signs and symptoms such as fever, tachycardia and tachypnea, leading to faster discharge from the hospital. Accordingly, TCS and LOS were reduced by about 20% in patients receiving adjunctive corticotherapy, corresponding to an absolute reduction of LOS of about one and a half day. Although the magnitude of this treatment effect is relatively small, it may have important economic implications given the high incidence of CAP. Considering its low cost, adjunctive corticotherapy might be cost-benefitial for institutions admitting CAP patients.

Adverse events seem to be infrequent and short-lasting. Although the absolute proportion of patients requiring insulin therapy during hospitalization was increased by 7.2% with adjunctive corticotherapy in our analysis, the number of patients with new insulin dependence at day 30 was very low in the largest available study.[16] Glucocorticoids might have some deleterious effects including rebound inflammation after treatment withdrawal and one study [35] reported an increased risk of late treatment failure among patients receiving adjunctive therapy. Nevertheless, these concerns were not confirmed in the larger STEP study.[16] Insufficient data was available to perform a meta-analysis on this outcome.

Our study has several potential limitations: First, although most studies were blinded, hyperglycemia among patients receiving adjunctive corticotherapy may have led to unblinding in some patients. Second, the type of corticosteroid administered, its dose and the duration of treatment varied among studies and the optimal corticosteroid regimen remains undefined. The median daily prednisone equivalent and treatment duration were 45mg and seven days in available studies. Third, CAP is a clinical and radiological syndrome with various etiologies, and the benefit of adjunctive corticotherapy might differ among pathogens. Since only a few studies reported separate results for microbiologically confirmed CAP, a subgroup analysis based on pathogens could not be performed. Finally, the benefit of adjunctive corticotherapy was exclusively evaluated among hospitalized patients and remains unknown among outpatients.

Our study also has several strengths. We performed a thorough literature search allowing identifying two studies[38–39] not included in previous systematic reviews and included more than 2000 patients, allowing a more precise estimation of the risks and benefits of adjunctive corticotherapy in CAP; the pertinence of including these older studies may be debated since anti-infectious and supportive care have evolved over the last decades. Nevertheless, the treatment effect estimate was not significantly different after excluding these older studies. Further, we were able to combine quantitative data regarding two relevant outcomes, LOS and TCS by using a statistical model adapted to the log-normal distribution of these outcomes and performed various sensitivity analyses, strengthening the validity of our findings. According to the available evidence, adjunctive corticotherapy benefits hospitalized CAP patients by a reduction of LOS and severe complications. Mortality may be reduced in patients with severe CAP. Further studies and meta-analyses based on patient characteristics are warranted to identify patients with the most favorable risk-benefit profile, and to determine the best regimen of adjunctive corticotherapy.

## Supporting Information

S1 File PRISMA Check List and detailed search strategy. (PDF)

S2 File Detailed results of secondary endpoints. (PDF)

S3 File Subgroup and sensitivity analyses. (PDF)

S4 File Publication bias. (PDF)
