# Supplementary material for: Adjunctive Corticotherapy for Community Acquired Pneumonia: A Systematic Review and Meta-Analysis
Source: PLoS One. 2015 Dec 7;10(12):e0144032. doi: 10.1371/journal.pone.0144032 (PMC4671611; doi:10.1371/journal.pone.0144032)

S2 File. Detailed results for secondary outcomes

• Length of Stay, Meta-analysis of geometric mean in each arm

	Control arm			Intervention arm		
**Study**	**Size**	**GM (95%CI)**	**Weight**	**Size**	**GM (95%CI)**	**Weight**
Confalonieri et al (2004)	23	17.4 (12.4 to 24.4)	11.6%	23	17.5 (14.7 to 20.9)	11.6%
Mikami et al (2007)	16	12.8 (10.5 to 15.4)	12.4%	15	10.2 (9.1 to 11.3)	12.6%
Snijders et al (2010)	109	6.8 (5.7 to 8.0)	12.5%	104	6.4 (5.4 to 7.6)	11.7%
Fernández-Serrano et al (2011)	22	12.5 (9.9 to 15.7)	12.3%	23	10.5 (9.4 to 11.9)	12.5%
Meijvis et al (2011)	153	7.7 (7.0 to 8.4)	12.8%	151	6.6 (6.2 to 7.1)	13.0%
Nafae et al (2013)	20	16.3 (16.2 to 16.5)	12.9%	60	9.0 (8.8 to 9.1)	13.2%
Blum et al (2015)	393	6.8 (6.3 to 7.3)	12.8%	392	6.0 (5.7 to 6.4)	13.0%
Torres et al (2015)	59	10.8 (9.6 to 12.2)	12.7%	61	10.5 (9.3 to 11.8)	12.5%
Pooled (random effects)		10.6 (7.4 to 15.3)			9.0 (7.6 to 10.7)	
I-squared		99.3%			97.8%	

The pooled geometric mean of the length of stay was 10.6 days (95%CI 7.4 to 15.3) in control arm and 9.0 days (95% 7.6 to 10.7) in intervention arm.

• Time to Clinical Stability, geometric Mean ratio, forest plot

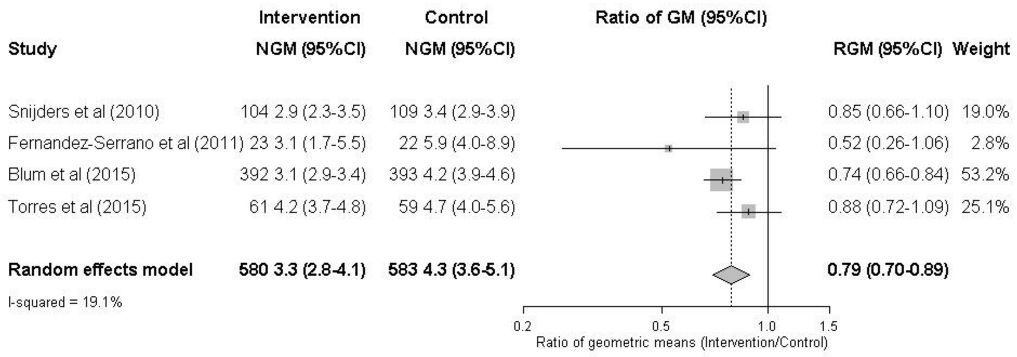

- ## Time to clinical stability, meta-analysis of geometric mean in each arm

	Control arm			Intervention arm		
**Study**	**Size**	**GM (95%CI)**	**Weight**	**Size**	**GM (95%CI)**	**Weight**
Blum et al (2015)	393	4.22 (3.85 to 4.64)	32.0%	392	3.13 (2.90 to 3.38)	34.6%
Fernández-Serrano et al (2011)	22	5.94 (4.00 to 8.85)	12.5%	23	3.11 (1.74 to 5.54)	8.4%
Snijders et al (2010)	109	3.36 (2.92 to 3.87)	28.6%	104	2.86 (2.33 to 3.52)	25.7%
Torres et al (2015)	59	4.72 (4.00 to 5.56)	26.9%	61	4.16 (3.65 to 4.75)	31.2%
Pooled (random effects)		4.25 (3.57 to 5.07)			3.34 (2.76 to 4.05)	
I-squared		78.7%			80.5%	

- ## Severe complications, forest plot

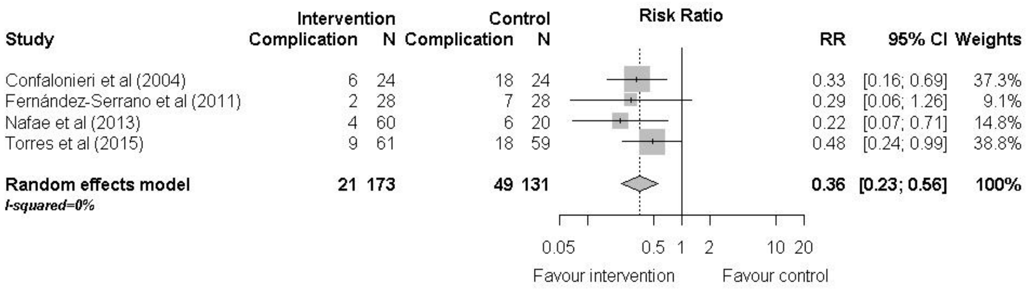

- ## Need for vasopressors, forest plot

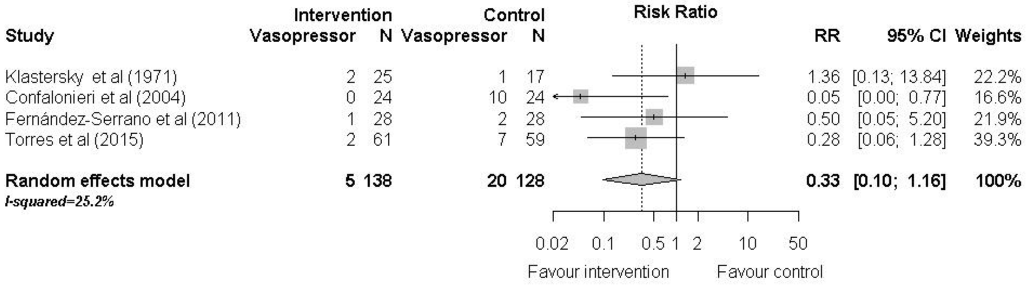

- ## Need for mechanical Ventilation, forest plot

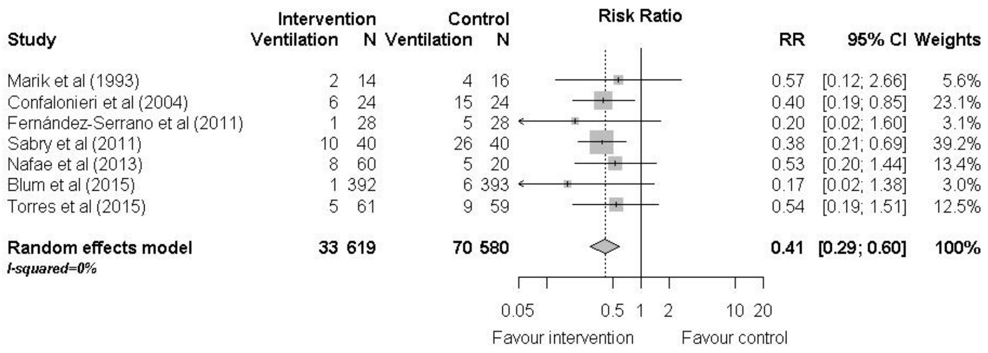

- ## Hyperglycemia, forest plot

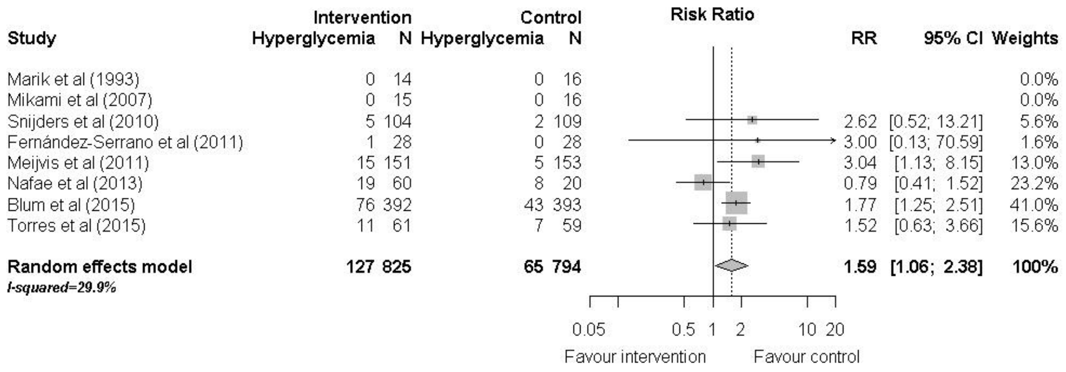

- ## Gastro-intestinal bleeding, forest plot

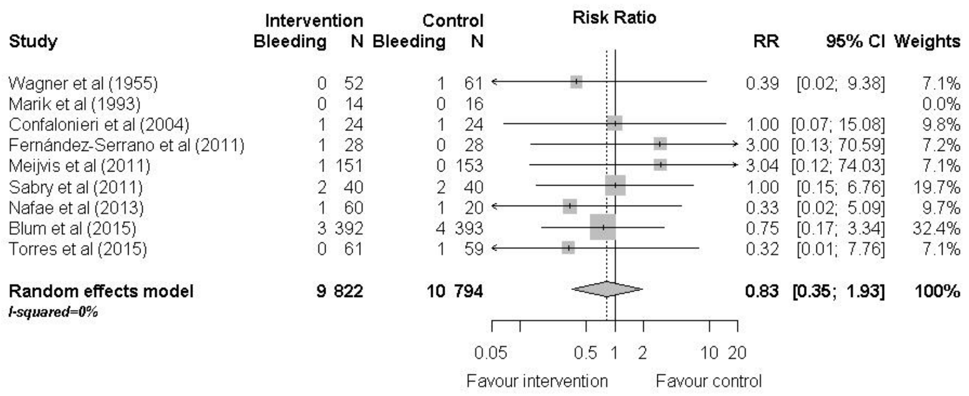

Supplement: S2 File — (PDF) [file pone.0144032.s002.pdf]
