# Supplementary material for: Adjunctive Corticotherapy for Community Acquired Pneumonia: A Systematic Review and Meta-Analysis
Source: PLoS One. 2015 Dec 7;10(12):e0144032. doi: 10.1371/journal.pone.0144032 (PMC4671611; doi:10.1371/journal.pone.0144032)
Supplement: S3 File — (PDF) [file pone.0144032.s003.pdf]

S3 File: Subgroup and sensitivity analyses

• Sub-groups analyses and meta-regressions for mortality

Sub-groups	Nb Studies	Pooled RR	p-value	I-square	Comparison p-value*
Severity					
Mixed severity	8	1.04 (0.66 to 1.64)	0.8765	43.5%	0.0672
SCAP	5	0.47 (0.23 to 0.96)	0.038	0.0%	
Dose					
≤50	7	1.11 (0.78 to 1.58)	0.5577	0.0%	0.1871
>50	6	0.54 (0.20 to 1.49)	0.233	67.4%	
Duration					
≤5 days	4	1.12 (0.56 to 2.24)	0.7566	38.0%	0.3473
>5 days	8	0.72 (0.40 to 1.31)	0.2796	51.9%	

*: p-value for between sub-groups comparisons

	Ratio de RR	p-values
Univariate meta-regression		
Mixed severity / SCAP	0.42 (0.17 to 1.09)	0.0744
High dose / Low dose	0.60 (0.25 to 1.47)	0.2676
Long / short duration	0.65 (0.24 to 1.73)	0.3895
Multivariable meta-regression		
High dose / Low dose	0.47 (0.18 to 1.22)	0.1227
Long / short duration	0.53 (0.20 to 1.40)	0.1991

• Leave-one-out sensitivity analyses

Mortality

Outcome	Study removed	Pooled RR (95%CI)	p-value	I-square (%)
**Mortality**	**All studies**	**0.84 (0.55 to 1.29)**	**0.4296**	**40.9%**
Mortality	Wagner et al (1955)	0.83 (0.53 to 1.29)	0.4039	45.8
Mortality	Bennett et al (1963)	0.76 (0.48 to 1.21)	0.2538	37.3
Mortality	Klastersky et al (1971)	0.77 (0.51 to 1.15)	0.1963	29.0
Mortality	McHardy et al (1972)	0.84 (0.54 to 1.33)	0.4657	45.3
Mortality	Marik et al (1993)	0.86 (0.56 to 1.33)	0.5079	43.7
Mortality	Confalonieri et al (2004)	0.91 (0.62 to 1.34)	0.6338	33.0
Mortality	Snijders et al (2010)	0.81 (0.51 to 1.29)	0.3797	45.9
Mortality	Fernández-Serrano et al (2011)	0.83 (0.54 to 1.29)	0.4128	45.8
Mortality	Meijvis et al (2011)	0.83 (0.51 to 1.33)	0.4356	45.6
Mortality	Sabry et al (2011)	0.9 (0.59 to 1.37)	0.6123	40.0
Mortality	Nafae et al (2013)	1.01 (0.7 to 1.45)	0.9772	20.6

Mortality	Blum et al (2015)	0.78 (0.48 to 1.26)	0.3117	44.8
Mortality	Torres et al (2015)	0.86 (0.54 to 1.36)	0.5165	43.9
**Mortality - MixedSeverity**	**All studies**	**1.04 (0.66 to 1.64)**	**0.8765**	**43.5%**
Mortality - MixedSeverity	Wagner et al (1955)	1.03 (0.63 to 1.67)	0.9190	51.6
Mortality - MixedSeverity	Bennett et al (1963)	0.94 (0.54 to 1.64)	0.8347	46.9
Mortality - MixedSeverity	Klastersky et al (1971)	0.92 (0.59 to 1.43)	0.7077	32.0
Mortality - MixedSeverity	McHardy et al (1972)	1.07 (0.65 to 1.77)	0.7913	49.5
Mortality - MixedSeverity	Snijders et al (2010)	1.02 (0.61 to 1.72)	0.9338	51.6
Mortality - MixedSeverity	Meijvis et al (2011)	1.06 (0.63 to 1.81)	0.8176	49.4
Mortality - MixedSeverity	Nafae et al (2013)	1.27 (0.91 to 1.78)	0.1548	0.0
Mortality - MixedSeverity	Blum et al (2015)	0.98 (0.56 to 1.72)	0.9474	51.3
**Mortality - SCAP**	**All studies**	**0.47 (0.23 to 0.96)**	**0.038**	**0.0%**
Mortality - SCAP	Marik et al (1993)	0.48 (0.22 to 1.04)	0.0636	3.1
Mortality - SCAP	Confalonieri et al (2004)	0.54 (0.26 to 1.12)	0.0993	0.0
Mortality - SCAP	Fernández-Serrano et al (2011)	0.44 (0.21 to 0.93)	0.0316	0.0
Mortality - SCAP	Sabry et al (2011)	0.52 (0.23 to 1.15)	0.1075	0.0
Mortality - SCAP	Torres et al (2015)	0.32 (0.11 to 0.93)	0.0358	0.0

GI Bleeding

Outcome	Study removed	Pooled RR (95%CI)	p-value	I-square (%)
**GI Bleeding**	**All studies**	**0.83 (0.35 to 1.93)**	**0.6633**	**0.0%**
GI Bleeding	Wagner et al (1955)	0.88 (0.36 to 2.12)	0.7717	0.0
GI Bleeding	Marik et al (1993)	0.83 (0.35 to 1.93)	0.6576	0.0
GI Bleeding	Confalonieri et al (2004)	0.81 (0.33 to 1.98)	0.6408	0.0
GI Bleeding	Fernández-Serrano et al (2011)	0.75 (0.31 to 1.80)	0.5154	0.0
GI Bleeding	Meijvis et al (2011)	0.75 (0.31 to 1.80)	0.5173	0.0
GI Bleeding	Sabry et al (2011)	0.79 (0.31 to 2.03)	0.6208	0.0
GI Bleeding	Nafae et al (2013)	0.91 (0.37 to 2.22)	0.8358	0.0
GI Bleeding	Blum et al (2015)	0.86 (0.31 to 2.43)	0.7800	0.0
GI Bleeding	Torres et al (2015)	0.89 (0.37 to 2.14)	0.7884	0.0

Hyperglycemia

Outcome	Study removed	Pooled RR (95%CI)	p-value	I-square (%)
**Hyperglycemia**	**All studies**	**1.59 (0.06 to 2.38)**	**0.0248**	**29.9%**
Hyperglycemia	Marik et al (1993)	1.59 (1.06 to 2.38)	0.0248	29.9
Hyperglycemia	Mikami et al (2007)	1.59 (1.06 to 2.38)	0.0248	29.9
Hyperglycemia	Snijders et al (2010)	1.54 (0.98 to 2.42)	0.0593	40.8
Hyperglycemia	Fernández-Serrano et al (2011)	1.58 (1.01 to 2.46)	0.0433	42.5
Hyperglycemia	Meijvis et al (2011)	1.45 (0.96 to 2.17)	0.0747	24.8
Hyperglycemia	Nafae et al (2013)	1.86 (1.38 to 2.51)	<0.0001	0.0
Hyperglycemia	Blum et al (2015)	1.56 (0.83 to 2.94)	0.1662	39.3
Hyperglycemia	Torres et al (2015)	1.63 (0.97 to 2.74)	0.0672	43.9

Mechanical ventilation

Outcome	Study removed	Pooled RR (95%CI)	p-value	I-square (%)
**Mechanical ventilation**	**All studies**	**0.41 (0.29 to 0.60)**	**<0.0001**	**0.0%**
Mechanical ventilation	Marik et al (1993)	0.41 (0.28 to 0.59)	<0.0001	0.0
Mechanical ventilation	Confalonieri et al (2004)	0.42 (0.28 to 0.63)	<0.0001	0.0
Mechanical ventilation	Fernández-Serrano et al (2011)	0.42 (0.29 to 0.61)	<0.0001	0.0
Mechanical ventilation	Sabry et al (2011)	0.43 (0.27 to 0.69)	0.0005	0.0
Mechanical ventilation	Nafae et al (2013)	0.40 (0.27 to 0.59)	<0.0001	0.0
Mechanical ventilation	Blum et al (2015)	0.43 (0.29 to 0.62)	<0.0001	0.0
Mechanical ventilation	Torres et al (2015)	0.40 (0.27 to 0.59)	<0.0001	0.0

Needs vasopressor

Outcome	Study removed	Pooled RR (95%CI)	p-value	I-square (%)
**Needs vasopressor**	**All studies**	**0.33 (0.10 to 1.17)**	**0.0847**	**25.2%**
Needs vasopressor	Klastersky et al (1971)	0.23 (0.07 to 0.80)	0.0211	9.0
Needs vasopressor	Confalonieri et al (2004)	0.46 (0.15 to 1.41)	0.1740	0.0
Needs vasopressor	Fernández-Serrano et al (2011)	0.29 (0.05 to 1.65)	0.1628	48.3
Needs vasopressor	Torres et al (2015)	0.35 (0.04 to 2.84)	0.3289	53.0

Severe complication

Outcome	Study removed	Pooled RR (95%CI)	p-value	I-square (%)
**Severe complication**	**All studies**	**0.36 (0.23 to 0.56)**	**<0.0001**	**0.0%**
Severe complication	Confalonieri et al (2004)	0.37 (0.21 to 0.66)	0.0006	0.0
Severe complication	Fernández-Serrano et al (2011)	0.37 (0.23 to 0.58)	<0.0001	0.0
Severe complication	Nafae et al (2013)	0.39 (0.24 to 0.63)	0.0001	0.0
Severe complication	Torres et al (2015)	0.30 (0.17 to 0.52)	<0.0001	0.0

Time to clinical stability

Study removed	Pooled RGM * (95%CI)	P-value	I-squared
Blum et al (2015)	0.848 (0.725 to 0.992)	0.0397	0.0%
Fernández-Serrano et al (2011)	0.794 (0.708 to 0.889)	<0.0001	18.4%
Snijders et al (2010)	0.775 (0.658 to 0.913)	0.0023	36.3%
Torres et al (2015)	0.755 (0.676 to 0.843)	<0.0001	1.5%

Length of stay

Study removed	Pooled RGM * (95%CI)	P-value	I-squared
Confalonieri et al (2004)	0.82 (0.64 to 1.06)	0.1272	97.3%
Mikami et al (2007)	0.82 (0.64 to 1.06)	0.2072	97.3%
Snijders et al (2010)	0.82 (0.64 to 1.06)	0.1383	97.2%
Fernández-Serrano et al (2011)	0.84 (0.65 to 1.09)	0.1798	97.3%
Meijvis et al (2011)	0.84 (0.64 to 1.09)	0.1839	96.7%
**Nafae et al (2013)**	**0.89 (0.84 to 0.94)**	**<0.0001**	**0.0%**
Blum et al (2015)	0.83 (0.64 to 1.08)	0.1623	95.9%
Torres et al (2015)	0.82 (0.64 to 1.06)	0.1228	96.9%

- ## Exclusion of older studies (Klastersky, Wagner, Benett and Mc hardy)

		Mantel-Haenszel - Random			
**Outcome**	**Nb studies**	**Pooled RR (95%CI)**	**p-value**	**I-squared**	
Mortality	9	0.64 (0.40 to 1.04)	0.0714	26.5%	
GI Bleeding	7	0.88 (0.36 to 2.12)	0.7708	0.0%	
Hyperglycemia	6	1.59 (1.06 to 2.38)	0.0248	29.9%	Unchanged
Mechanical ventilation	7	0.41 (0.29 to 0.60)	<0.0001	0.0%	Unchanged
Needs Vasopressor	3	0.23 (0.07 to 0.80)	0.0211	9.0%	
Severe complication	4	0.36 (0.23 to 0.56)	<0.0001	0.0%	Unchanged

• Exclusion of studies with higher risk of bias

	Not good quality (JADAD≤3)	Good quality (JADAD>3)	p for comparison good vs not good quality)
**Mortality**			
Nb studies	4	9	
Pooled RR	0.64 (0.19 to 2.13), I2=75%, p=0.47	1.03 (0.73 to 1.44), I2=0%, p=0.87	0.46
**Mortality in mixed severity studies**			
Nb studies	3	5	
Pooled RR	0.77 (0.18 to 3.34), I2=81%, p=0.72	1.19 (0.82 to 1.74), I2=0%, p=0.35	0.57
**Mortality in SCAP studies**			
Nb studies	1	4	
Pooled RR	0.33 (0.07 to 1.55), I2=NA, p=0.16	0.52 (0.23 to 1.15), I2=0%, p=0.11	0.62
**GI Bleeding**			
Nb studies	2	6	
Pooled RR	0.70 (0.15 to 3.33), I2=0%, p=0.65	0.89 (0.32 to 2.44), I2=0%, p=0.82	0.80
**Hyperglycemia**			
Nb studies	2	6	
Pooled RR	0.79 (0.41 to 1.52), I2=NA, p=0.48	1.86 (1.38 to 2.51), I2=0%, p<0.0001	0.02
**Mechanical ventilation**			
Nb studies	2	5	
Pooled RR	0.42 (0.25 to 0.69), I2=0%, p=0.0007	0.41 (0.24 to 0.69), I2=0%, p=0.0009	0.95
**Needs Vasopressor**			
Nb studies	1	3	
Pooled RR	1.36 (0.13 to 13.84), I2=NA, p=0.80	0.23 (0.07 to 0.80), I2=9%, p=0.02	0.19
**Severe complication**			
Nb studies	1	3	
Pooled RR	0.22 (0.07 to 0.71), I2=NA, p=0.01	0.39 (0.24 to 0.63), I2=0%, p=0.0001	0.38
**Time to clinical stability**			
Nb studies	0	4	
Pooled GMR	/	0.79 (0.70 to 0.89), I2=19%, p<0.0001	NA
**Length of stay**			
Nb studies	2	6	
Pooled GMR	1.54 (1.07 to 2.21), I2=91%, p=0.02	1.12 (1.05 to 1.19), I2=0%, p=0.0005	0.09

- ## Treatment effect estimate according to year of publication

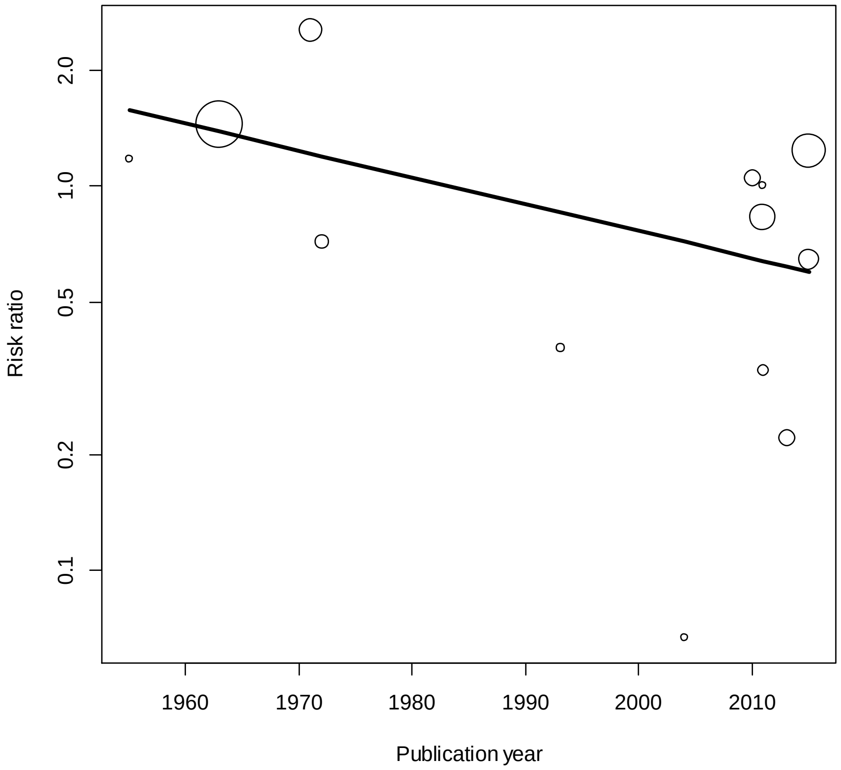

The logarithm of the relative risk is reduced by 0.16 (se 0.09) for every ten years (**p=0.0626**).
