# Supplementary material for: Adjunctive Corticotherapy for Community Acquired Pneumonia: A Systematic Review and Meta-Analysis
Source: PLoS One. 2015 Dec 7;10(12):e0144032. doi: 10.1371/journal.pone.0144032 (PMC4671611; doi:10.1371/journal.pone.0144032)
Supplement: S4 File — (PDF) [file pone.0144032.s004.pdf]

S4 File. Publication bias

Publication bias, funnel plots

- **Length of stay**

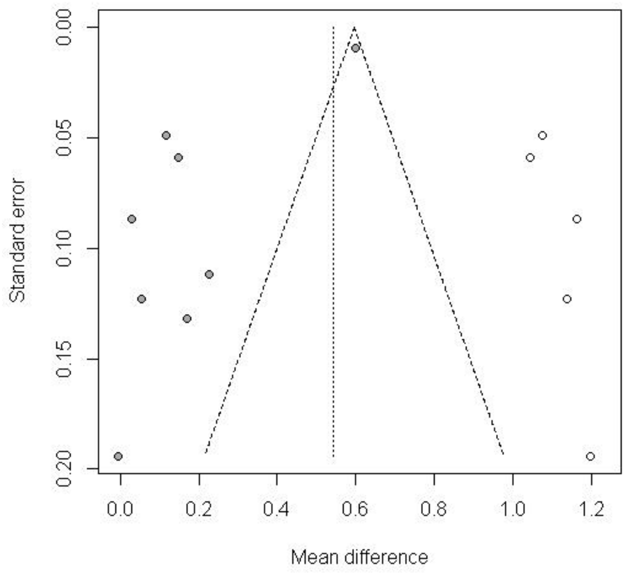

- **Severe complication**

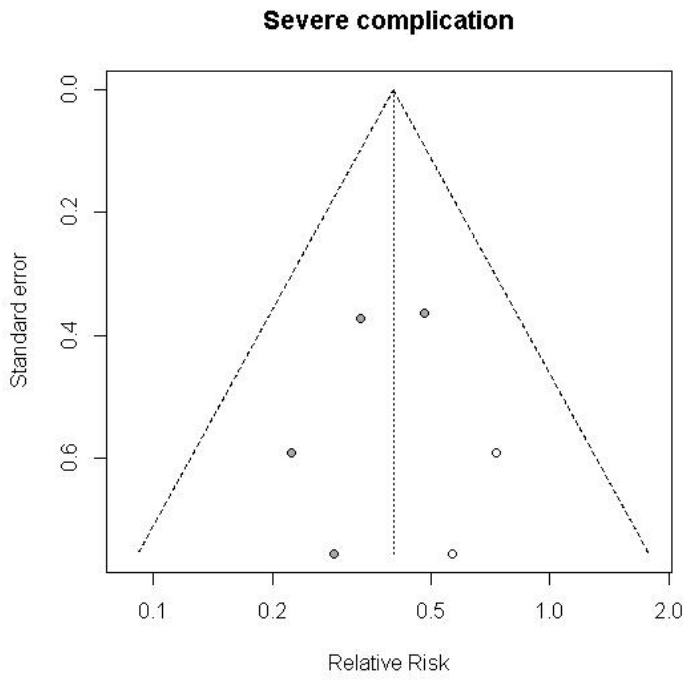

- **Need for vasopressors**

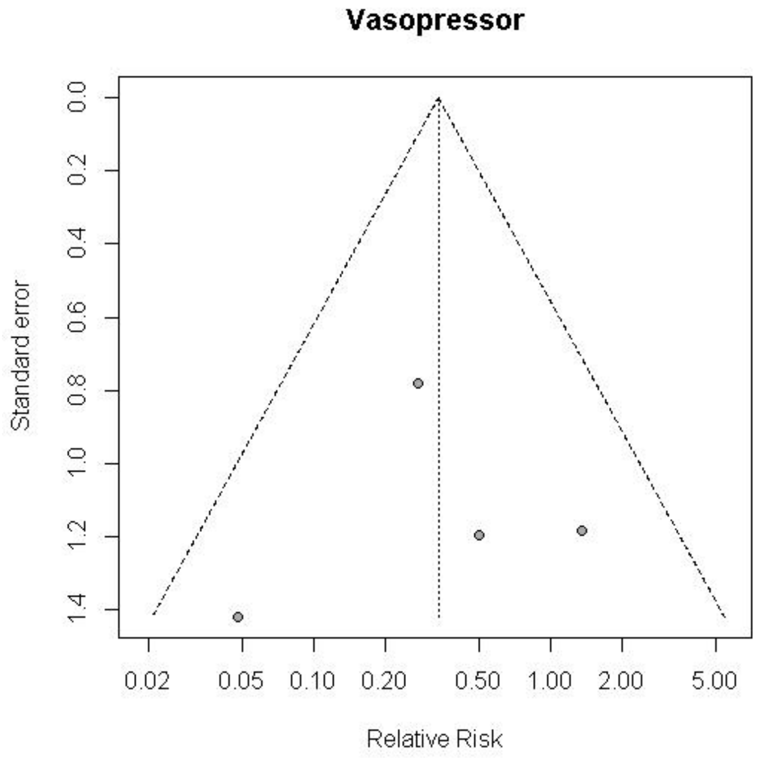

- ## Need Mechanical ventilation

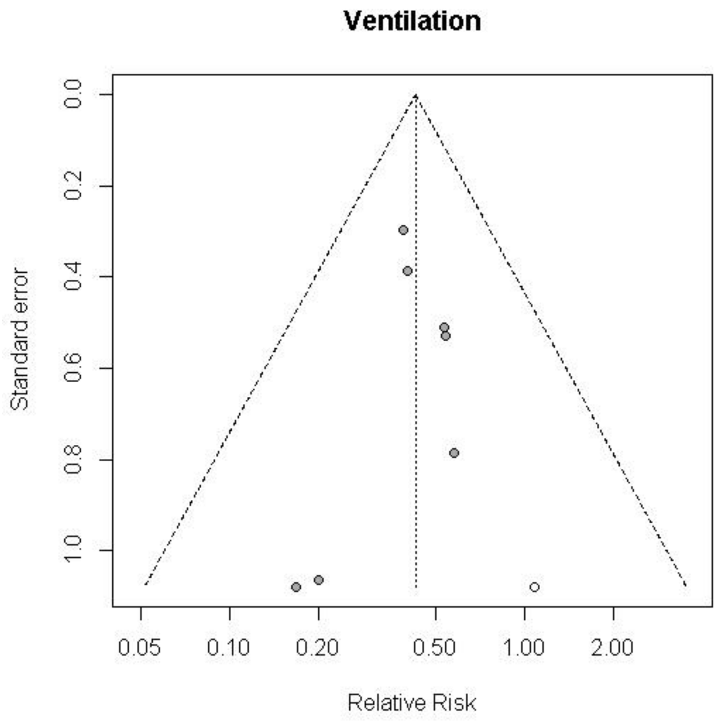

- ## Hyperglycaemia

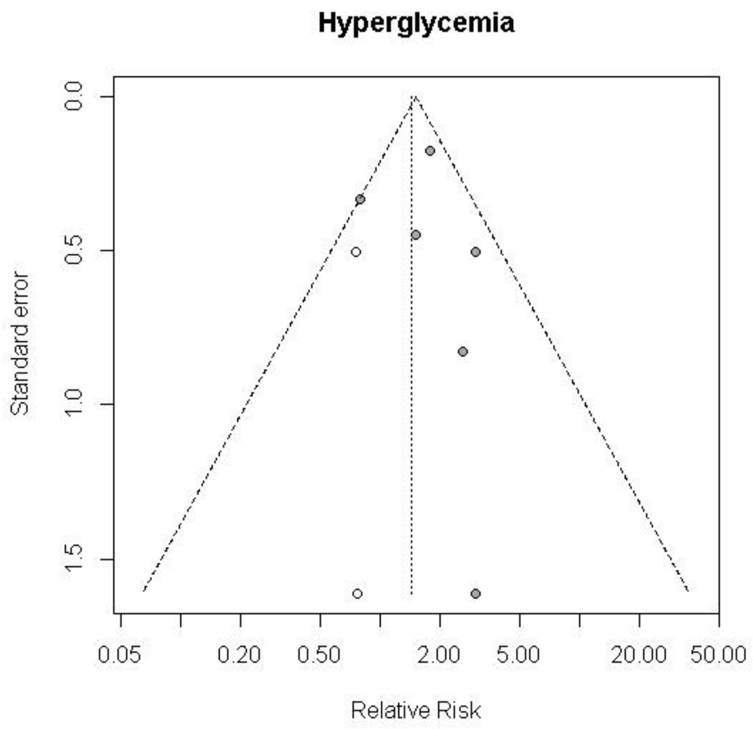

- **Gastro-intestinal bleeding**

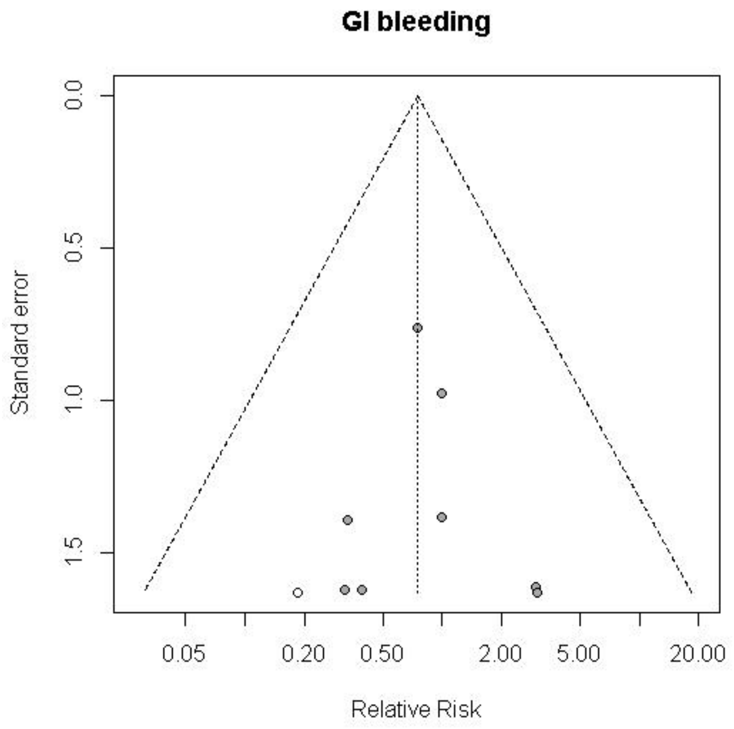

Appendix Q. Publication bias, Trim & Fill, Egger's test

Outcome	Trim & Fill Pooled RR (95%CI)	p-value	Egger's test	N missing	Side missing
Mortality	1.24 (0.78 to 1.97)	0.3687	0.0517	5	Right
Mixed severity	1.32 (0.78 to 2.22)	0.2950	NA *	2	Right
SCAP	0.54 (0.27 to 1.08)	0.0815	NA *	1	Right
GI Bleeding	0.75 (0.33 to 1.70)	0.4924	NA *	1	Left
Hyperglycemia	1.45 (1.00 to 2.09)	0.0482	NA *	2	Left
Mechanical ventilation	0.43 (0.30 to 0.61)	<0.0001	NA *	1	Right
Needs Vasopressor	0.33 (0.10 to 1.17)	0.0847	NA *	0	
Severe complication	0.40 (0.27 to 0.60)	<0.0001	NA *	2	Left

*: not enough studies
